# Microbiological exploration of the Cueva del Viento lava tube system in Tenerife, Canary Islands

**DOI:** 10.1111/1758-2229.13245

**Published:** 2024-04-21

**Authors:** Sara Gutierrez‐Patricio, Jorge R. Osman, José Luis Gonzalez‐Pimentel, Valme Jurado, Leonila Laiz, Alfredo Laínez Concepción, Cesareo Saiz‐Jimenez, Ana Zélia Miller

**Affiliations:** ^1^ Instituto de Recursos Naturales y Agrobiología de Sevilla (IRNAS‐CSIC) Sevilla Spain; ^2^ Instituto de Geología Económica Aplicada (GEA) Universidad de Concepción Concepción Chile; ^3^ Laboratorio HERCULES Universidade de Évora Évora Portugal; ^4^ Grupo de Espeleología de Tenerife Benisahare Tenerife Islas Canarias Spain

## Abstract

Cueva del Viento, located in the Canary Islands, Spain, is the Earth's sixth‐longest lava tube, spanning 18,500 m, and was formed approximately 27,000 years ago. This complex volcanic cave system is characterized by a unique geomorphology, featuring an intricate network of galleries. Despite its geological significance, the geomicrobiology of Cueva del Viento remains largely unexplored. This study employed a combination of culture‐dependent techniques and metabarcoding data analysis to gain a comprehensive understanding of the cave's microbial diversity. The 16S rRNA gene metabarcoding approach revealed that the coloured microbial mats (yellow, red and white) coating the cave walls are dominated by the phyla *Actinomycetota*, *Pseudomonadota* and *Acidobacteriota*. Of particular interest is the high relative abundance of the genus *Crossiella*, which is involved in urease‐mediated biomineralization processes, along with the presence of genera associated with nitrogen cycling, such as *Nitrospira*. Culture‐dependent techniques provided insights into the morphological characteristics of the isolated species and their potential metabolic activities, particularly for the strains *Streptomyces* spp., *Paenarthrobacter* sp. and *Pseudomonas* spp. Our findings underscore the potential of Cueva del Viento as an ideal environment for studying microbial diversity and for the isolation and characterization of novel bacterial species of biotechnological interest.

## Graphical abstract

Cueva del Viento, located in the Canary Islands, Spain, is the world's sixth‐longest lava tube, formed 27,000 years ago. Despite its significant geological importance, the cave's geomicrobiology has yet to be explored. This study aims to uncover the composition of the bacterial community using culture‐dependent methods and metabarcoding data analysis. The dominant phyla in the cave sites studied are *Actinomycetota*, *Pseudomonadota* and *Acidobacteriota*. The strains cultured from these sites hold potential for the discovery of novel compounds with biotechnological applications.

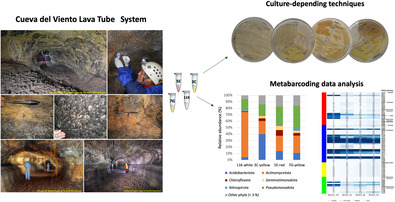

## INTRODUCTION

Caves, tunnels and mines are important subterranean resources, each with distinct origins resulting from either natural geological processes or anthropic activities. While tunnels and mines have significant economic implications for society, cave ecosystems offer unique natural laboratories for studying the ecology and evolution of subsurface microbial communities, due to the temporal and spatial isolation of their biota (Poulson & White, 1969). Among cave types, karstic caves, formed by the dissolution of carbonate rocks, have received the most attention (de Waele et al., 2011). In contrast, volcanic caves, mainly represented by lava tubes, have been comparatively less studied (White, 2005).

Lava tubes, formed due to the differential cooling of lava during volcanic eruptions, are located in volcanic regions worldwide. In the United States, numerous lava tubes have been explored, particularly in Hawaii, where volcanic activity is ongoing (Léveillé & Datta, 2010; Northup & Lavoie, 2015; Riquelme, Rigal, et al., 2015). Other locations that contain lava tubes and have been the subject of geomicrobiological studies include the Canary Islands, Spain (Gonzalez‐Pimentel et al., 2018; Gonzalez‐Pimentel et al., 2021; Gonzalez‐Pimentel et al., 2022), Easter Island, Chile (Miller et al., 2014), Italy (Nicolosi et al., 2023), the Galapagos Islands, Ecuador (Miller et al., 2020; Miller et al., 2022), Iceland (Kopacz et al., 2022) and the Azores, Portugal (Hathaway et al., 2014; Riquelme, Marshall Hathaway, et al., 2015).

These cave systems harbour complex microbial communities that often form biofilms, which coat the walls and ceilings of these subterranean environments (Gonzalez‐Pimentel et al., 2018; Riquelme, Marshall Hathaway, et al., 2015; Riquelme, Rigal, et al., 2015). These biofilms exhibit a range of sizes, from small millimetric colonies to extensive microbial mats. In addition, they can display different colours, including grey, yellow, orange, pink, red and white, not only in lava tubes but also in karstic caves (Gonzalez‐Pimentel et al., 2021; Saiz‐Jimenez et al., 2011). Through processes such as rock fracture and water percolation, microorganisms from the surface eventually reach the caves, where only those with adaptable metabolisms can successfully establish long‐term colonization within the subsurface environment. Lavoie (Lavoie et al., 2017) observed that, when comparing the microbial communities of the lava tubes and those of the surface soil, the same taxonomic groups were reproduced at the phyla level in both scenarios. However, they only shared 11.2% of Operational Taxonomic Units (OTUs). There are two dominant bacterial groups, which alternate in first and second positions, according to different studies based on DNA analysis to determine diversity in lava tubes: *Actinomycetota* and *Pseudomonadota* (Gonzalez‐Pimentel et al., 2018; Hathaway et al., 2014; Riquelme, Marshall Hathaway, et al., 2015). In general, a wide microbial diversity can be found in caves, encompassing numerous bacterial phyla, with *Pseudomonadota*, *Actinomycetota*, *Acidobacteriota*, *Nitrospirota* and *Bacillota* as the most abundant groups (Biagioli et al., 2023; De Mandal et al., 2017; Saiz‐Jimenez, 2012). In addition, minority phyla such as *Verrucomicrobiota*, *Planctomycetota*, *Gemmatimonadota* and *Chloroflexota* and other phyla, considered as microbial dark matter (He, 2023; Lok, 2015) or belonging to rare biosphere (Hershey & Barton, 2018; Lynch & Neufeld, 2015; Pascoal et al., 2021; Saw, 2021) have also been detected. The rare biosphere comprises microorganisms that can only be identified using deep‐sequencing NGS (Next Generation Sequencing) approaches (Sogin et al., 2006).

The results obtained using molecular and traditional tools show disparity in terms of the major groups found in subterranean environments. With traditional isolation techniques, the most abundant microorganisms were members of the *Actinomycetota* and *Bacillota* phyla (Dominguez‐Moñino et al., 2021; Groth et al., 1999; Urzì et al., 2010), while the molecular approaches revealed the existence of different and specific phyla promoted by the microenvironment (De Mandal et al., 2017; Gonzalez‐Pimentel et al., 2018; Saiz‐Jimenez, 2012).

The processes of metabolic adaptation of microorganisms to very specific conditions make their cultivation in the laboratory difficult, which has promoted the use of molecular techniques to achieve a complete knowledge of the biodiversity of subterranean environments, including groups of microorganisms unknown until the introduction of these techniques (Saiz‐Jimenez, 2012). The combination of microbiological, molecular biology and geochemical techniques has allowed a wide development of geomicrobiological issues (Gabriel & Northup, 2013). These multidisciplinary approaches have revealed that chemolithoautotrophic microorganisms are of major importance within the trophic chain of subterranean niches, since they act as primary producers, further supporting the development of heterotrophic microorganisms. They, together with the entry of nutrients from outside through water percolation, airflow and the intrusion of plant roots located on the surface, provide the necessary nutritional supply for their development in caves (Jobbagy & Jackson, 2001; McCulley et al., 2004; Saiz‐Jimenez & Hermosin, 1999; Sarbu et al., 1996; Schenk & Jackson, 2002). It should also be noted the presence of sulfur, organic carbon and nitrogen associated with the chemical composition of the basaltic substrate in the lava tubes, which favours the presence of sulfur and nitrogen oxidizing and reducing microorganisms (Chen et al., 2009; Hathaway et al., 2014).

Here, using a multiproxy approach based on electron microscopy, culture‐dependent methods and NGS technologies, we aimed to bridge gaps in our understanding of microbial communities in lava tubes and provide the first study into the microbiota dwelling on the walls of one of the world's largest lava tube, the Cueva del Viento in Tenerife (Canary Islands, Spain). This volcanic cave system is the sixth‐longest lava tube in the world (Gulden, 2022). Cueva del Viento, whose name derives from the strong air currents circulating inside the tubes, depicts a unique geomorphology, related to the network of galleries distributed along three superimposed levels (Oromí & Socorro, 2021). Numerous studies indicate a great faunal richness in this cavity (Láinez et al., 2014; Oromí & Socorro, 2021; Socorro Hernández et al., 2010). It harbours 36 troglobionts, with nine species exclusive to this cave system. Additionally, the remains of numerous extinct vertebrates, including the Tenerife giant lizard (*Gallotia goliath*) and the Tenerife giant rat (*Canariomys bravoi*), have been discovered in Cueva del Viento. Yet, the studies on the microorganisms of Cueva del Viento are very limited (Gutiérrez Patricio, 2016; Gutiérrez Patricio et al., 2022), contrasting with the existing information on other lava tubes from the Canary Islands (Gonzalez‐Pimentel et al., 2018; Gonzalez‐Pimentel et al., 2021; Riquelme, Marshall Hathaway, et al., 2015; Riquelme, Rigal, et al., 2015). This study provides novel insights into the microbial ecology of Cueva del Viento, a lava tube system renowned for its remarkable and distinct structures. Cueva del Viento, with its distinctive geomorphology and strong internal air currents, presents an ideal site for advancing our knowledge of cave microbial ecology. This study contributes to filling the knowledge gaps in the microbial ecology of lava tubes, particularly in the context of Cueva del Viento's unique environmental conditions and microbial diversity.

## EXPERIMENTAL PROCEDURES

### Sampling sites

**FIGURE 1:**
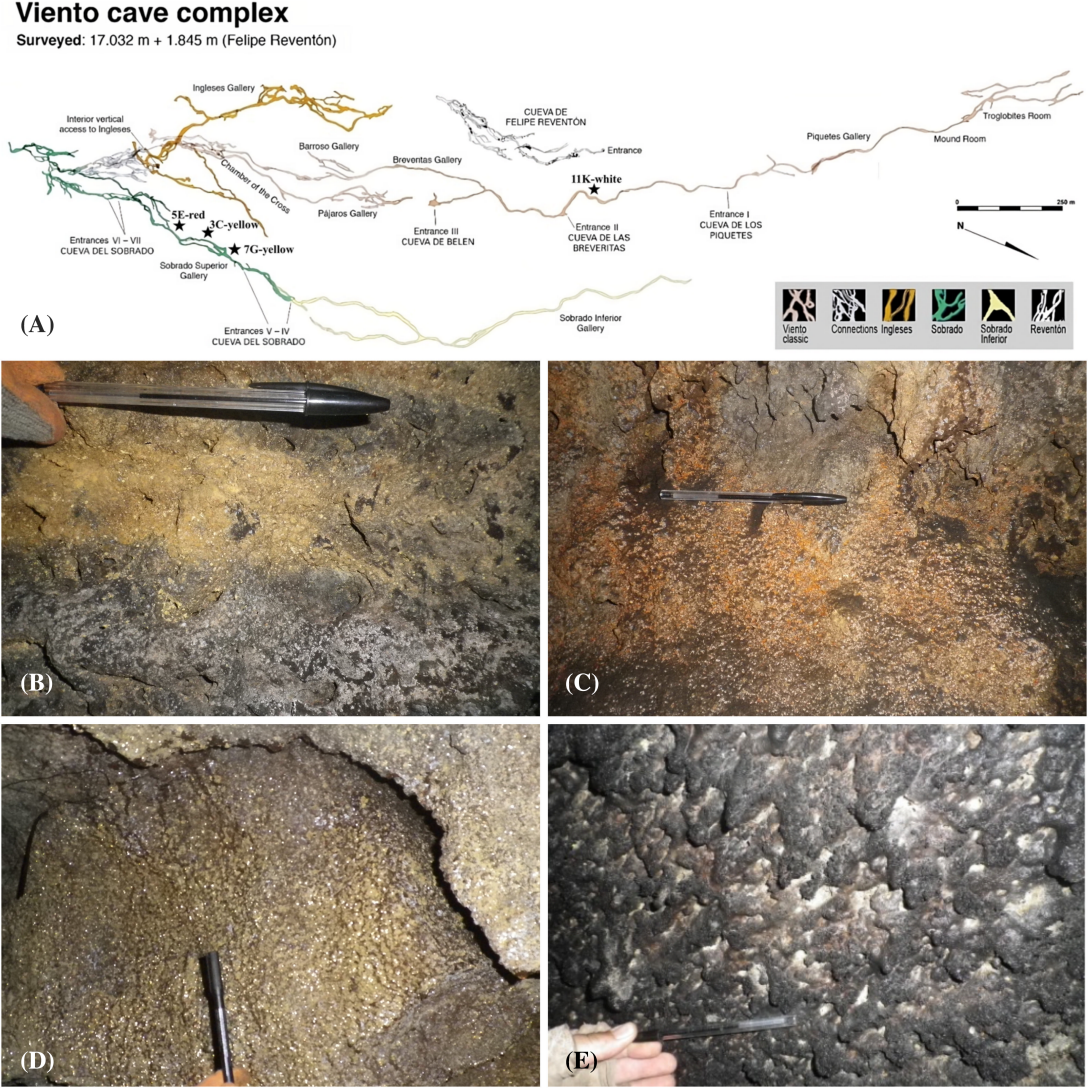
Map of Cueva del Viento lava tube system (A, adapted from Oromí and Socorro [2021]) and localization of the samples 3C‐yellow (B), 5E‐red (C), 7G‐yellow (D) and 11K‐white (E).

The Canary Islands archipelago (Spain), composed of eight main islands of volcanic origin, is located in the eastern Atlantic Ocean on the coast of southern Morocco. In the center of the insular chain is Tenerife, which is the largest island (2034 km^2^), highest (3714 m at the peak of Teide), and most diverse, both in ecosystems and in animal and plant species. The climate of this area is mild (annual mean 15.1°C) and moderately humid (Oromí & Socorro, 2021). The town of *Icod de Los Vinos*, located in the northwest of the island, has a high density of lava tubes, in which Cueva del Viento is located (Oromí, 2018; Oromí & Socorro, 2021). This cave (Figure 1A), formed 27,000 years ago and with approximately 18,500 m, was formed within lava composed of basalt and plagioclase‐rich pāhoehoe lava flows. The basaltic rocks consist of well‐formed and twinned crystals of calcium‐rich feldspars (specifically anorthite), as well as mesocrysts of clinopyroxenes and olivine. The dark‐coloured minerals often appear as a hypocrystalline to a vitreous matrix with round vesicles (Carracedo, 2008).

Coloured microbial mats were observed and sampled along three different sections of the lava tube. Four sampling sites exhibiting extensive microbial mats with characteristic colours (yellow, red and white) coating the walls and ceiling of Cueva del Viento were collected for microscopy, DNA‐based analysis, and culture‐dependent techniques. Three replicate samples were obtained aseptically per sampling site by scraping the microbial mats with a sterile scalpel, stored in sterile tubes at 4°C, and immediately cultured upon arrival at the laboratory. Samples for DNA extraction were kept at −80°C.

The collected microbial mats were named 3C‐yellow (Figure 1B), 5E‐red (Figure 1C) and 7G‐yellow (Figure 1D) for the yellow and red biofilm samples located in the cave section not open to visitors, called *Sobrado Superior* Gallery (tube B); and 11K‐white for the whitish‐coloured biofilms located in the *Breveritas Inferior* Gallery (Figure 1E).

### Field emission scanning electron microscopy observations

Field emission scanning electron microscopy (FESEM) was used to obtain high‐resolution images of the microbial mat samples, allowing for the visualization of their surface features, microbial cell morphology and structural characteristics. Samples were air‐dried and directly mounted on sample stubs, coated with gold–palladium using a sputtering technique. A JEOL JSM‐7001F microscope with an Oxford EDS detector (Oxford Microbeam, Oxford, UK) was used with the secondary electron (SE) detection mode and an acceleration voltage of 15 kV.

### Culture‐dependent methods

For culture techniques, aliquots of the microbial mat samples were resuspended in a sterile 0.85% (w/v) NaCl solution and subsequently seeded on nutrient agar (Difco), tryptone‐soy agar (TSA, Difco) and TSA diluted at 0.3% with 3% iron, 3% magnesium and 3% silicon salts. All samples were incubated at 30°C for 7 weeks. After this time, the bacterial colonies were selected according to their morphological characteristics.

The identification of the bacterial isolates was carried out using molecular biology techniques based on the analysis of the 16S rRNA gene. DNA was extracted using the protocol described by Griffiths (Griffiths et al., 2000). The 16S rRNA gene was amplified by PCR using the primers 616F 5′‐AGAGTTTGATYMTGGCTCAG‐3′ (Juretschko et al., 1998) and, 1510R 5′‐GGCTACCTTGTTACGACTT‐3′ (Echigo et al., 2005).

PCR reactions were performed in a FlexCycler thermocycler (Analytik Jena AG, Germany) using the following conditions: 94°C for 2 min; 35 cycles of 94°C for 20S, 55°C for 20S, 72°C for 2 min and the final step of 72°C for 10 min. Positive PCR products were purified using the JetQuick PCR Purification Spin Kit (GenoMed Inc., Leesburg, FL) according to the manufacturer's instructions and stored at −20°C for further analysis.

The PCR‐purified products were sequenced at the Secugen Laboratory (CSIC, Madrid, Spain), and the sequences obtained were edited using the BioEdit Sequence Alignment Editor program (version 7.0.4.1; Hall, 1999).

Identification of 16S rRNA gene sequences was performed using the EZBioCloud database and the National Center for Biotechnology Information (NCBI) database using the BLASTN algorithm. The sequences were deposited in the NCBI GenBank database (http://www.ncbi.nlm.nih.gov/genbank/) with the accession numbers LN867256‐LN867289 and LN881695‐LN881702.

### Metabarcoding data analysis

Total genomic DNA was extracted from approximately 250 mg of each sample using the DNeasy PowerSoil Kit (Qiagen, Germany) according to the manufacturer's instructions and quantified using a Qubit 4.0 fluorometer (Invitrogen). The V3‐V4 hypervariable region of the prokaryotic 16S rRNA gene was amplified by PCR reactions using 341F/805R primer pairs and sequenced using a MiSeq platform to generate paired 300 bp reads, according to Macrogen's Illumina Miseq Reagent Kit v3 library preparation protocol (Seoul, Korea).

The raw reads were processed using QIIME2 v.2023.2 (Bolyen et al., 2019). Following the guidelines provided by DADA2, an Amplicon Sequence Variants (ASVs) table was generated (Callahan et al., 2016). The SILVA database, version 138.1, was employed for taxonomic classification, applying a similarity cutoff of 97% (Quast et al., 2013). To statistically assess the biodiversity within the samples, both the Shannon and Pielou indices were calculated, which measure richness and evenness, respectively (Pielou, 1966; Shannon, 1948). Samples were also compared based on their taxonomic abundances with Venn diagrams using the Venny 2.1 tool (Oliveros, 2015). In addition, a Principal Component Analysis (PCA) was performed using Statgraphics Centurion XVIII software for the simultaneous ordination of different microbial communities at the genus taxonomic level (higher than 0.5% abundance) and the identification of the dominant microbial communities of each biofilm sample (Palma et al., 2024).

The bioinformatics tool PICRUSt2 was used to analyse the potential functionality of the microbial communities focusing on the main pathways involved in some of the major biogeochemical cycles: nitrogen, carbon and sulfur (Douglas et al., 2020). This was achieved through the prediction of the KEGG orthologs and their abundance in each sample (Kyoto Encyclopedia of Genes and Genomes; https://www.genome.jp/kegg/pathway.html). Taxa with an NSTI score below 2 were not considered.

## RESULTS AND DISCUSSION

### Morphological characteristics of the coloured microbial mats

The Cueva del Viento lava tube system has a colourful and diversified variety of microbial mats, ranging in colour from white to yellow and red (Figure 1), as frequently reported in lava tubes worldwide (Hathaway et al., 2014; Northup et al., 2011; Riquelme, Marshall Hathaway, et al., 2015; Riquelme, Rigal, et al., 2015). These microbial mats comprise small scattered colonies, varying in size from 1 to 2 mm to several cm with irregular edges that cover extensive areas of Cueva del Viento's walls and ceiling.

**FIGURE 2:**
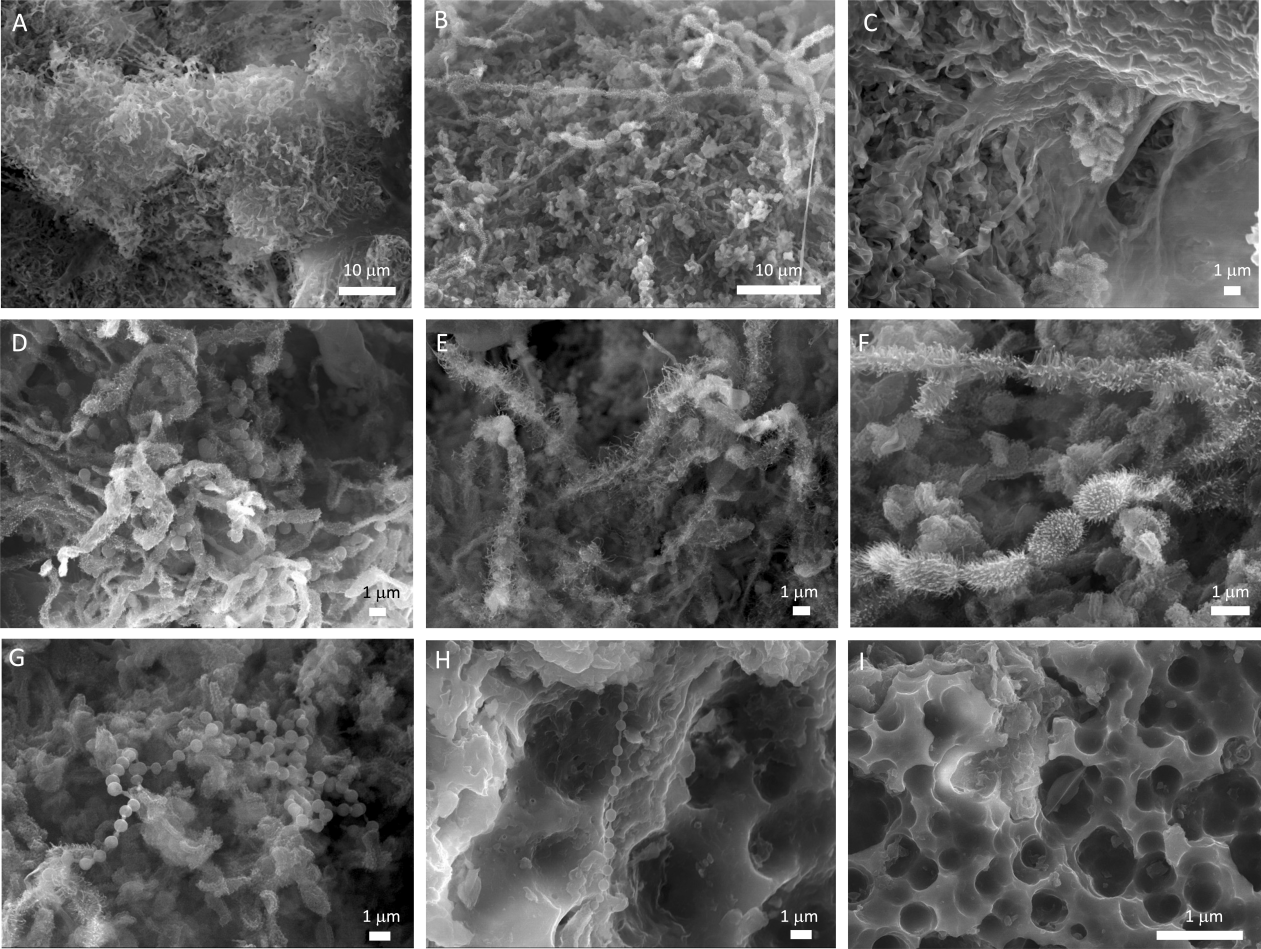
Representative field emission scanning electron microscope images of the coloured microbial mat samples collected from the Cueva del Viento lava tube system, showing a wide range of microbial morphologies. (A, B) Dense tangled masses of filamentous structures characteristic of *Actinomycetota*‐rich microbial mats; (C) aggregations of bacterial filaments embedded in a gel‐like substance of extracellular polymeric substances; (D) Branched filaments with the characteristic morphology of *Actinomycetota* and coccoid cells; (E) Detailed image of *Actinomycetota*‐like filaments with hairy ornamentation; (F) Rod‐shaped cells arranged in rows with spiny appendages; (G, H) Bacterial filaments resembling beads‐on‐a‐string and (I) Microboring or cell‐sized etch pits on the siliceous substrate.

Although the microbial mats differed in appearance, colonies with distinct colours, similar microbial features, and biofilm structure are found at the microscopic scale (Figure 2). The FESEM examinations revealed dense tangle masses of microbial cells embedded in slimy matrices of extracellular polymeric substances (EPS) for all the samples (Figure 2A–C). These biofilms contain a diverse array of microbial morphologies, including filamentous and coccoidal structures, branching patterns, and complex three‐dimensional architectures, which are consistent with *Actinomycetota*‐like mats found in lava tubes (Gonzalez‐Pimentel et al., 2018; Riquelme, Marshall Hathaway, et al., 2015; Riquelme, Rigal, et al., 2015). At higher magnification, most of the individual filamentous cells comprising these biofilms display surfaces with hairy ornamentation and rods arranged in rows with spiny appendages (Figure 2D–F). One of the intriguing microbial features found in the samples from Cueva del Viento is beads‐on‐a‐string (Figure 2G, H), resembling those filaments reported by Northup (Northup et al., 2011) in lava tubes from Hawai'i, New Mexico and Azores. The FESEM also revealed interactions of the microbial mats with siliceous minerals. Coccoid‐shaped etched pits indicative of mineral dissolution processes by biological activity are observed associated with microbial cells (Figure 2I). This microboring is commonly due to the release of organic acids by bacterial cells, which dissolve the underlying siliceous substrate (Miller et al., 2014). These microbe‐mineral interactions leave behind biosignatures that provide evidence of microbial activity functioning and can be crucial in understanding life on the early Earth or for the search for microbial life in planetary caves (Westall et al., 2015; Wynne et al., 2022).

### Bacterial isolates

Forty‐two bacterial strains were isolated by culture techniques from the microbial mats collected in the Cueva del Viento lava tube system (Table S1). The mainly isolated strains belonged to the phyla *Actinomycetota* (43%) and *Bacillota* (41%) in agreement with the findings from other worldwide caves (Dominguez‐Moñino et al., 2021; Groth et al., 1999; Urzì et al., 2010). The phylum *Pseudomonadota* (12%) was observed in the 7G‐yellow and 11K‐white biofilm samples. The phylum *Bacteroidota* (5%) was also found in this last sample, constituting the most diverse sample obtained by culture methods.

The genus *Streptomyces* comprised 44% of the bacterial strains isolated from Cueva del Viento and was detected in all samples except for the 7G‐yellow biofilm (Table S1). This genus is widely distributed and abundant in soils, including compost (Kämpfer, 2012), and has also been found in volcanic caves and karst caves (Gutiérrez Patricio, 2016; Riquelme et al., 2017). The genus *Streptomyces* is formed by 721 species of chemoorganotrophic organisms, capable of degrading complex and recalcitrant plant and animal matter. Some species are pathogenic to animals and humans, while others are phytopathogens (Kämpfer, 2012). Three species of this genus were identified in the 3C‐yellow biofilm sample (*Streptomyces rhizosphaerihabitans*, *S. benahoarensis* and *S. apricus*). Notably, *Streptomyces benahoarensis* was originally isolated from a lava tube on La Palma (Canary Islands, Spain). This species plays an important role in the resistome, contributing to the synthesis of active compounds, and is known for producing antimicrobial substances, such as linocin‐M18 and curamycin (Gonzalez‐Pimentel et al., 2022). *S. apricus*, on the other hand, was isolated from soils sampled in Wisconsin (USA) (Hariharan et al., 2022). The presence of these *Streptomyces* species in Cueva del Viento highlights that they might be involved in the degradation of organic matter inside the cave. Moreover, their ability to produce antimicrobial compounds suggests a role in inhibiting the growth of certain pathogens and competitors, thus influencing the microbial diversity within the cave system.

Members belonging to the *Arthrobacter*, *Paenarthrobacter* and *Micrococcus* genera were also isolated. The *Arthrobacter ginkgonis* species was only isolated from the 11K‐white biofilm sample. This species was originally isolated from *Ginkgo biloba* root, a native tree from China (Cheng et al., 2017). The genus *Paenarthrobacter* was found in the 7G‐yellow (three strains) and the 11K‐white (two strains) biofilm samples. All the isolated strains were classified as *P. nicotinovorans*. This species is characterized by the degradation of nicotine, atrazine and other triazine herbicides (Aislabie et al., 2005; Busse, 2016; Kodama et al., 1992). Also, three strains of the genus *Micrococcus*, which is characterized as chemoorganotrophs with strictly respiratory metabolism, were isolated and identified as *M. luteus*, a species originally isolated from mammalian skin.

The *Bacillota* phylum was the second most abundant and the first in terms of biodiversity. It was also recently isolated from other European volcanic caves (work in progress). Nine strains of *Bacillus*, consisting of a large number of heterogeneous bacteria widely distributed in the soil and subsoil, were isolated from these microbial mats (Table S1). Some species are used in industrial applications such as the synthesis of pharmaceuticals and chemical compounds, or food production as is the case of *B. siamensis* (detected in the 7G‐yellow and 5E‐red biofilm), initially isolated from a popular Thai food condiment (Poo‐khem) (Sumpavapol et al., 2010). Other isolated species were *B. mycoides* (7G‐yellow biofilm), *B. atrophaeus* and *B. tequilensis* (5E‐red biofilm). Three *Bacillus* strains were also isolated but could not be identified at the species level. These bacteria might be involved in the degradation of organic matter, influencing cave microbial community structure through competitive and symbiotic interactions. Their presence is evidence of high input of organic matter into the cave.

The *Peribacillus*, *Psychrobacillus* and *Rossellomorea* genera were isolated from the 7G‐yellow biofilm, while the *Metabacillus* and *Sutcliffiella* genera were isolated from the 11K‐white biofilm. The *Psychrobacillus* genus is characterized by being psychrotolerant; only one strain corresponding to *P. soli* was isolated that can degrade oil, and it was initially isolated from an oil‐contaminated soil near a gas station (Pham et al., 2015). The isolated *Rossellomorea* and *Metabacillus* genera are commonly found in marine environments, such as *R. vietnamensis* (Gupta et al., 2020; Noguchi et al., 2004) and *M. rhizolycopersici* (Ma et al., 2022).

*Pseudomonas* was the most abundant genus, belonging to the *Pseudomonadota* phylum, isolated from the 7G‐yellow and 11K‐white biofilms. Species of this genus are characterized by a very versatile metabolism, so they present important biotechnological properties such as the strain isolated from the 7G‐yellow biofilm *P. laurylsulfatiphila*, which is characterized by degrading sodium dodecylsulfate (SDS). This strain was originally isolated from the peat soil of a wastewater treatment plant (Furmanczyk et al., 2018). *Pseudomonas lalkuanensis* and *P. yangonensis* were isolated from the 11K‐white sample and were reported to be previously isolated from wound samples and a bacterial consortium of polluted soil enriched for e‐waste remediation, respectively (Thorat et al., 2020; Tohya et al., 2020). Another isolated genus from the white biofilm was *Pseudoxanthomonas*. Members belonging to this genus were reported to reduce arsenic and nitrate (Mohapatra et al., 2018; Thierry et al., 2004).

**FIGURE 3:**
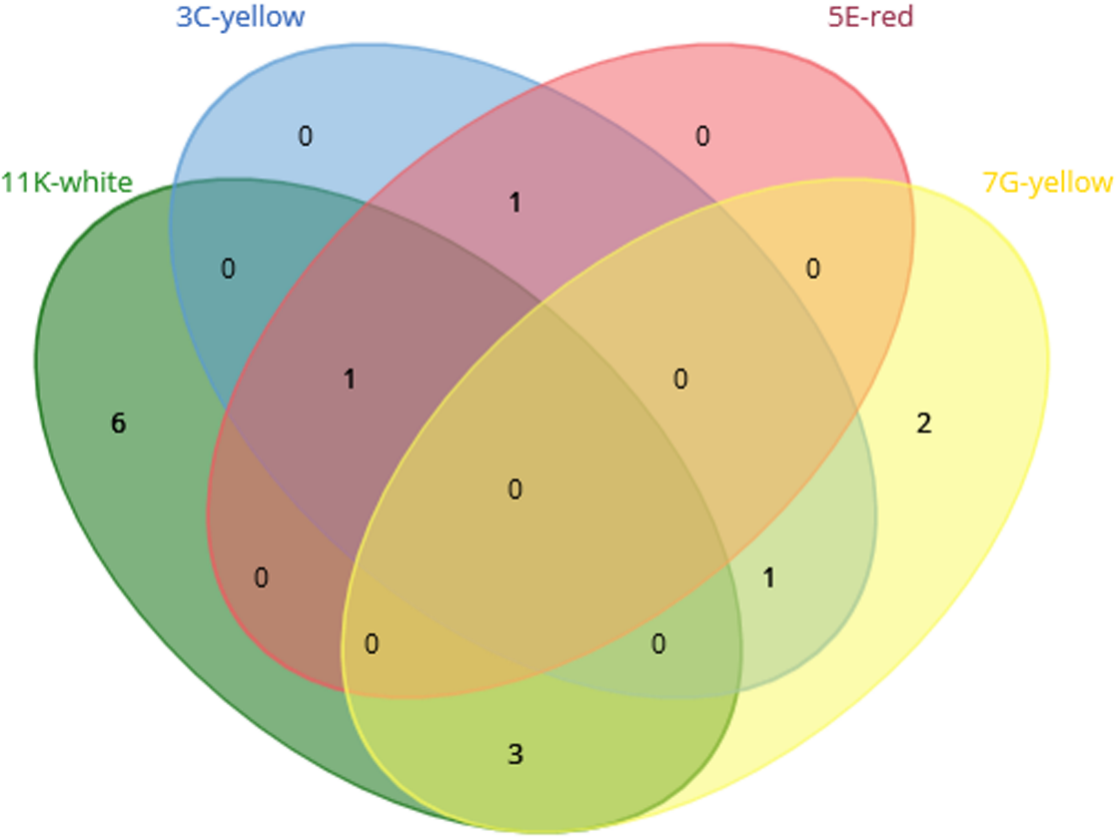
Venn diagram representing the distribution of the number of the isolated genera detected by culture‐dependent methods in each sample and the overlap among the samples.

The *Bacteroidota* phylum is formed by chemoorganotrophic bacteria and it was only detected in the 11K‐white biofilm. The isolated species *Chryseobacterium candidae* and *Flavihumibacter fluminis* were previously isolated from *Candida* yeast (Indu et al., 2020) and river silt (China) (Guo et al., 2023), respectively. Both strains deserve interest because this phylum is infrequently isolated by traditional culturing techniques. Despite presenting values higher than 97% similarity they could be good candidates for description as new species (Rosselló‐Móra & Amann, 2001), since the 16S rRNA gene value of 97% is not a strict cutoff value. To compare the distribution of unique isolated bacterial genera, we applied a Venn diagram (Figure 3). Interestingly, no common genera were found across all the samples. The 11K‐white biofilm exhibited the highest number of unique isolated genera, with six in total, followed by the 7G‐yellow biofilm with two unique genera. The *Pseudomonas*, *Micrococcus* and *Paenathrobacter* genera were shared between these two biofilms. The 5E‐red and 3C‐yellow biofilms, on the other hand, did not exhibit any unique genera among the observed isolates.

In general, the bacteria isolated in these samples might utilize the organic matter present in this cave, contributing to the formation and dynamics of biofilms. These biofilms can interact with the siliceous minerals as observed by FESEM. In addition, these bacteria could possess the ability to degrade complex organic substrates and synthesize bioactive substances, highlighting their ecological and biotechnological potential. It is important to note that the culture media used in this study may not effectively target many of the microorganisms present in this lava tube system, such as oligotrophic and chemolithotrophic microorganisms, due to the limitations of the media employed (Duncan et al., 2021).

### Bacterial communities composition

A total of 791,108 raw DNA sequences (average length 450 bp) were obtained after Illumina Miseq sequencing of PCR amplified V3‐V4 16S rRNA genes from total extracted DNA from the four studied microbial mats (Table 1). After the sequences trimming process, a total of 448,830 high‐quality sequences remained. The ASVs ranged from 452 to 1813. The 5E‐red biofilm shows the highest richness among the samples, followed by the 7G‐yellow biofilm that contains 1474 ASVs. The Chao estimator ranged from 1855.66 in the 5E‐Red biofilm to 452.0 in the 3C‐yellow sample. The diversity indices (Shannon and Simpson indices) showed that the 5E‐red, 7G‐yellow and 3C‐yellow mats contain relatively similar diversity and that the 11K‐white biofilm was 2‐fold lower in diversity estimation compared to the other samples, which is reflected in the bacterial composition determined by sequencing.

**TABLE 1:** Number of sequences, richness and diversity for each coloured biofilm sample.

Sample	High‐quality sequences				
	Number of sequences	ASVs	Chao1	Shannon index	Simpson index
11K‐White	116,262	1209	1234.93	4.53	0.730
3C‐Yellow	135,974	452	452.0	7.83	0.992
5E‐Red	101,088	1813	1855.66	8.34	0.987
7G‐Yellow	95,506	1474	1489.5	7.91	0.983

**FIGURE 4:**
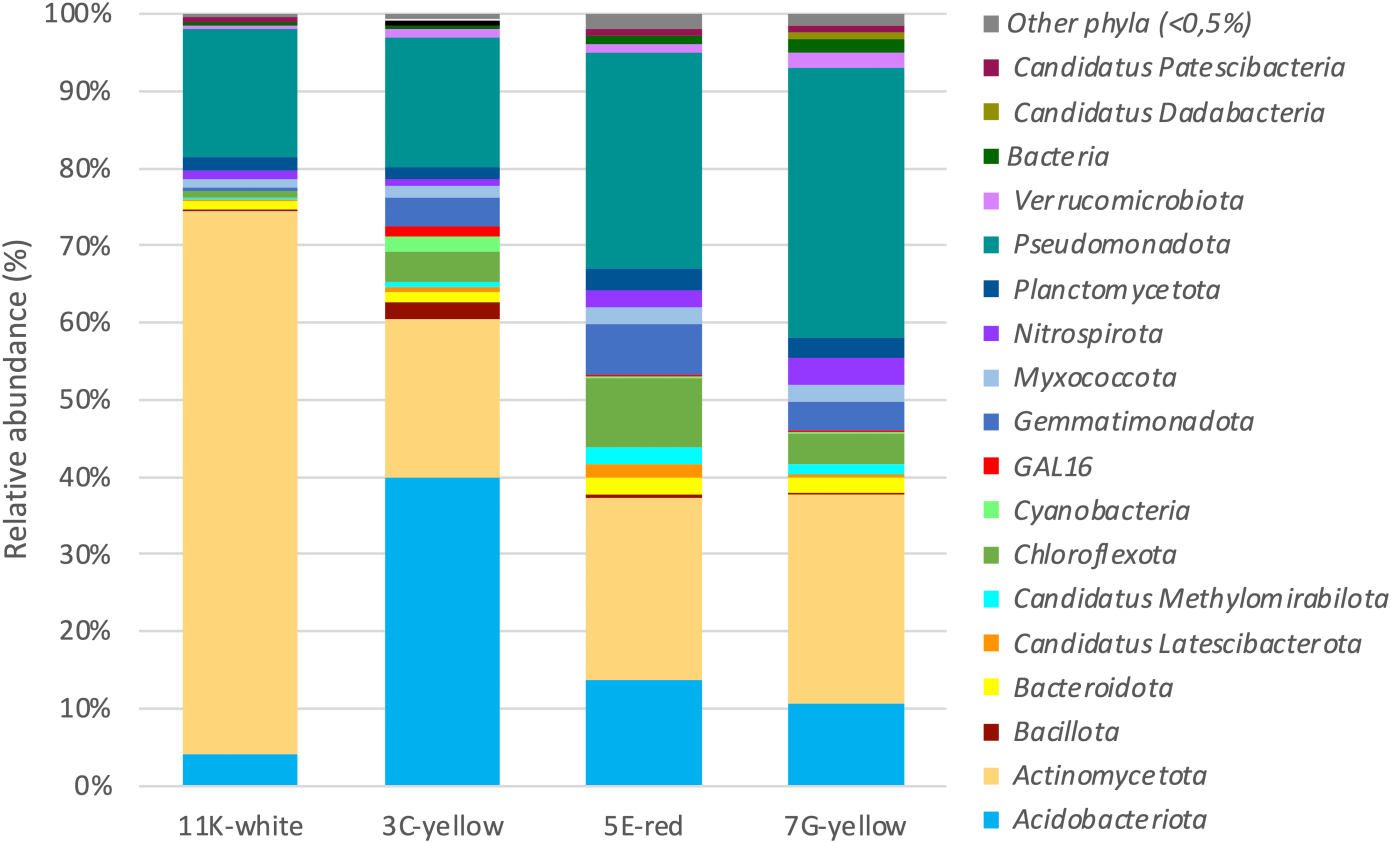
Bacterial composition at the taxonomic rank of phylum for the coloured biofilm samples detected by metabarcoding analysis based on the relative abundance. “Other phyla” are those below the cutoff of 0,5%.

Thirty‐seven prokaryotic phyla were identified, but their distribution showed different percentages for each biofilm, depending on the coloration (Figure 4). The 11K‐white biofilm was composed of 26 bacterial phyla with relative abundances <1%, the 7G‐yellow biofilm 20 phyla, the 5E‐red biofilm 16, and the 3C‐yellow biofilm 13. All the biofilms showed a similar number of phyla (12–13) with relative abundance >1%, except in the 11K‐white biofilm with only six phyla. In total, the phyla with relative abundances >1% represented over 99% of all the microbial mats, except in the 7G‐yellow and 5E‐red which amounted to 98.30% and 97.86%, respectively.

The phyla *Actinomycetota, Pseudomonadota* and *Acidobacteriota* dominated in the four biofilms, with relative abundances altogether of 91.23% for the 11K‐white, 77.19% for the 3C‐yellow, 72.54% for the 7G‐yellow and 65.18% for the 5E‐red biofilms. It was noteworthy the high presence of *Actinomycetota* in the 11K‐white biofilm (70.45%), *Acidobacteriota* in the 3C‐yellow biofilm (39.83%) and *Pseudomonadota* in the 7G‐yellow biofilm (34.95%). Other phyla with relative abundances >5% were *Chloroflexota* and *Gemmatimonadota* in the 5E‐red biofilm. Phyla with relative abundances >1% (*Bacteroidota, Cyanobacteriota, Bacillota*, GAL15, *Candidatus* Latescibacterota, *Candidatus* Methylomirabilota, *Myxococcota, Nitrospirota, Planctomycetota* and *Verrucomicrobiota*) retrieved from the biofilms were commonly found in microbial communities from lava tubes (Gonzalez‐Pimentel et al., 2021; Miller et al., 2020; Saiz‐Jimenez et al., 2011). In addition, most of the low‐abundance phyla (<0.1%) included *Armatimonadetes*, *Bdellovibrionota*, MBNT15, NB1‐j and RCP2–54 among other phyla, which are considered part of a rare biosphere (Lynch & Neufeld, 2015; Saw, 2021) and particularly members of a cave rare biosphere (Hershey & Barton, 2018).

Yellow‐coloured biofilms are widely distributed across volcanic and limestone caves (Gonzalez‐Pimentel et al., 2018; Miller et al., 2022; Porca et al., 2012; Saiz‐Jimenez et al., 2011), as well as in Cueva del Viento (Figure 1). Martin‐Pozas (Martin‐Pozas et al., 2023) reported that there are various types of yellow biofilms in caves worldwide, exhibiting differences in both morphology and bacterial compositions. Interestingly, the authors also revealed that within the same cave, the yellow biofilms displayed different bacterial compositions (Martin‐Pozas et al., 2023). Remarkably, the two yellow biofilms from Cueva del Viento showed different phyla abundances and were different from those karstic caves, where *Pseudomonadota* was the most abundant phylum and *Actinomycetota* was less abundant than *Acidobacteriota*.

**FIGURE 5:**
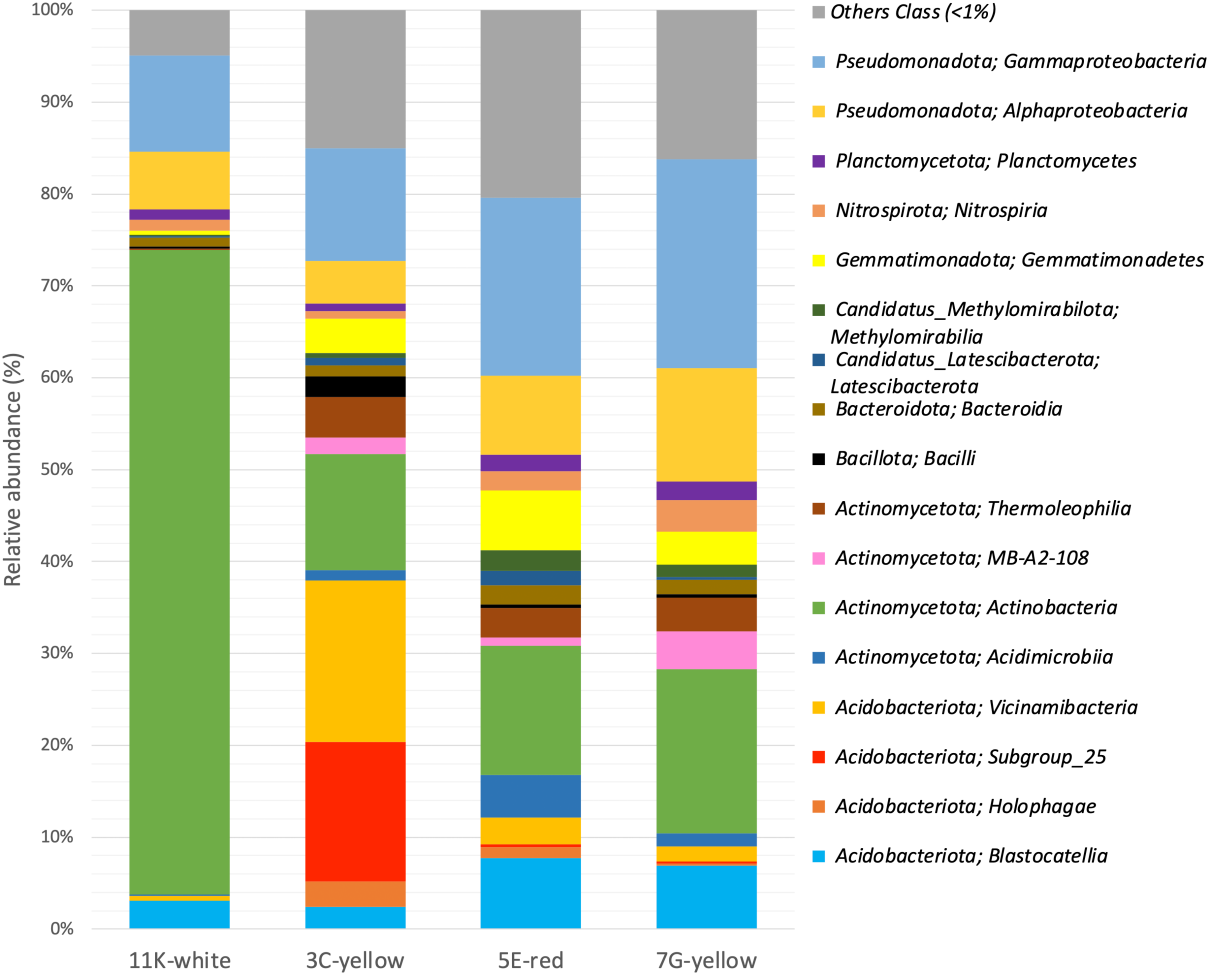
Bacterial composition at the taxonomic rank of class for the coloured biofilm samples detected by metabarcoding analysis based on the relative abundance. Classes below the cutoff of 1% are shown as “other class”.

The distribution of classes in the four biofilms was remarkably different (Figure 5), with the outstanding dominance of *Actinobacteria* in the 11K‐white biofilm (70.08% of relative abundance) while in the other three coloured biofilms ranged between 17.84% and 12.67%. The second most abundant class was *Gammaproteobacteria*; in this case, the 11K‐white biofilm attained the lower relative abundance (10.47%), and the 3C‐yellow and 7G‐yellow biofilms reached a relative abundance of 12.24% and 22.63%, respectively, while the 5E‐red biofilm attained 19.35%. The high abundances of *Actinobacteria* and *Gammaproteobacteria* in lava tubes were already reported by other authors (Gonzalez‐Pimentel et al., 2021; Nicolosi et al., 2023; Riquelme, Marshall Hathaway, et al., 2015).

The two most abundant classes were *Vicinamibacteria* and subdivision 25 of *Acidobacteriota* (Figure 5). These classes of *Acidobacteriota* were particularly abundant in the 3C‐yellow biofilm (17.53–15.17%). In the 7G‐yellow biofilm, *Alphaproteobacteria* reached a relative abundance of 12.33%. The *Vicinamibacteria* class was previously reported in lava tubes and is abundant in soils. The occurrence of subdivision 25 seems to be less frequent, both in caves and soils (de Chaves et al., 2019; Gonzalez‐Pimentel et al., 2021; Janssen, 2006; Miller et al., 2020; Nicolosi et al., 2023; Prescott et al., 2022). Other *Acidobacteriota* with relative abundance ranging from 2.42% to 7.71% was *Blastocatellia*, most abundant in 5E‐red and 7G‐yellow biofilms. With relative abundances around 3% were *Holophagae*. This class was also recorded in a lava tube from La Palma Island (Gonzalez‐Pimentel et al., 2021), although with lower relative abundances than in Cueva del Viento and Hawaiian caves (Prescott et al., 2022).

Other classes of *Actinomycetota* were observed in Cueva del Viento in addition to *Actinobacteria*: *Acidimicrobiia, Thermoleophilia*, and MB‐A2‐108, with varying relative abundances in each biofilm (Figure 5). *Actinobacteria* and *Acidimicrobiia* were present in another lava tube with similar or lower relative abundances (Gonzalez‐Pimentel et al., 2021). All four classes of *Actinomycetota* were also recorded in Hawaiian caves (Prescott et al., 2022).

*Alphaproteobacteria* exhibited relative abundances between 4.64% and 12.33%. Generally, this class is less abundant than *Gammaproteobacteria* in lava tubes (Gonzalez‐Pimentel et al., 2021; Prescott et al., 2022). The class *Gemmatimonadetes* was relatively abundant in the yellow and 5E‐red biofilms (3.62–6.48%), but scarce in the 11K‐white biofilm (0.43%). This class appeared frequently in diverse caves although with low abundances (Jurado et al., 2020; Paun et al., 2019; Reboleira et al., 2022).

Other groups were represented by five *Chloroflexota* classes (*Anaerolineae*, *Chloroflexotaa*, *Ktedonobacteria*, JG30‐KF‐CM66 and KD4‐96). These classes showed abundances of 1%–2% in the 5E‐red biofilm, and *Anaerolineae* only in the 7G‐yellow biofilm, but other classes showed relative abundances of <1% or were absent in the biofilms. *Nitrospiria*, *Bacilli*, *Bacteroidia*, *Planctomycetes*, *Latescibacterota*, *Methylomirabilia*, *Cyanobacteriia*, GAL15, *Polyangia*, bacteriap25 (*Myxococcota*) and *Verrucomicrobiae*, presented different abundances in the biofilms (0–3.46%). All these classes are frequently reported in volcanic and karstic caves (Addesso et al., 2021; Gonzalez‐Pimentel et al., 2021; Jurado et al., 2020; Lavoie et al., 2017; Prescott et al., 2022).

**FIGURE 6:**
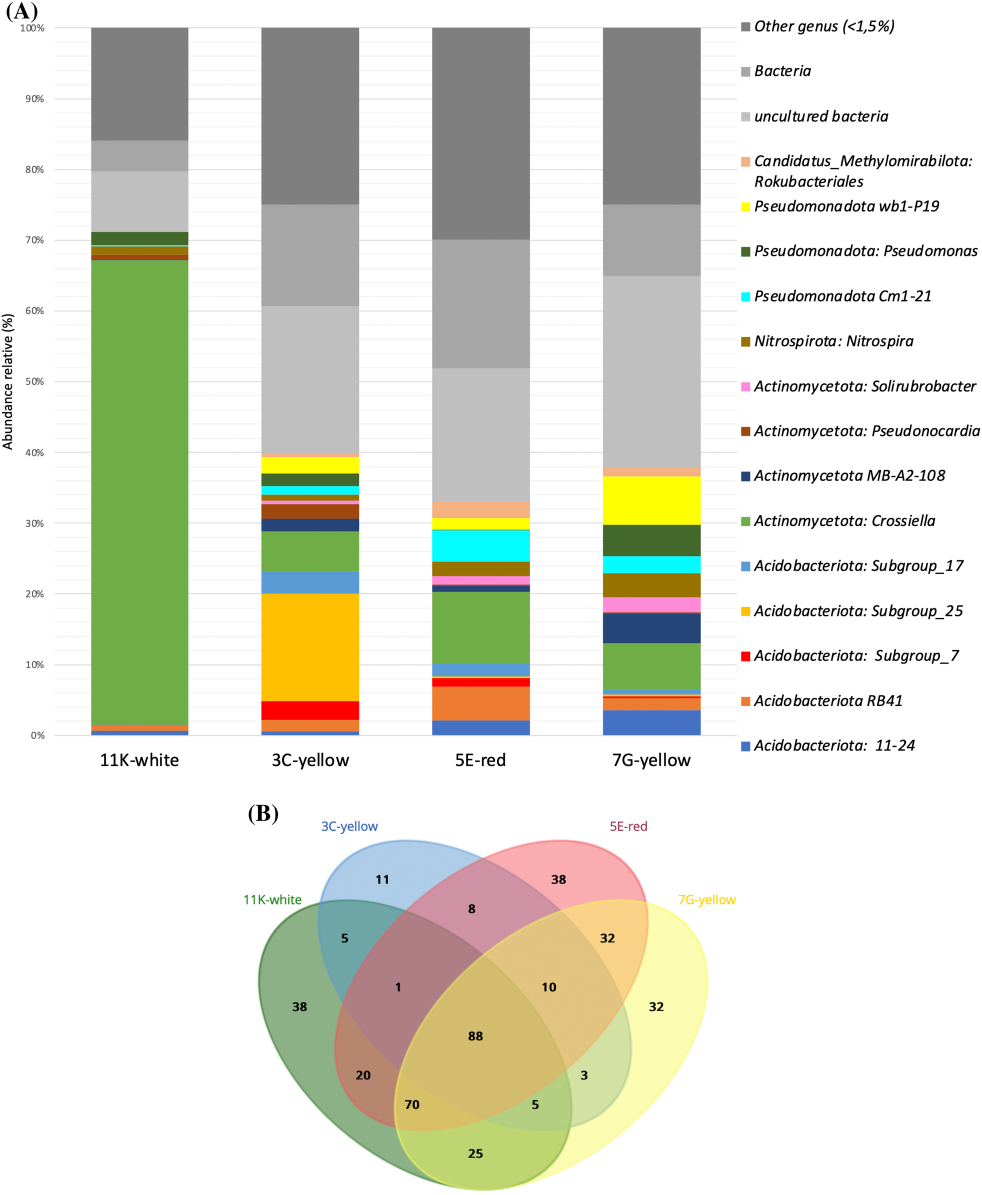
(A) Bacterial composition at the taxonomic rank of genus for the coloured biofilm samples detected by metabarcoding analysis based on the relative abundance. (B) Venn diagram representing the distribution of the number of genera detected by metabarcoding analysis in each sample and the overlap among the samples.

Figure 6A shows the 26 bacterial genera with relative abundance >1% for all the biofilms. In addition, 86 genera with relative abundances <0.1% were retrieved. It is worth noting the high relative abundance of *Crossiella* in the 11K‐white biofilm (65.63%). Similar abundances in lava tubes of Mount Etna (Sicily, Italy) were reported by Nicolosi (Nicolosi et al., 2023). There, *Crossiella* abundances ranged between 62.5% and 77.6%. Lower *Crossiella* abundances were found in moonmilk and other deposits from La Palma Island lava tubes (6.7%–38.9%). In addition, *Crossiella* was retrieved in moonmilk from karstic caves (24.00%–27.06%) and yellow biofilms (5.31%–7.87%) (Martin‐Pozas et al., 2023). Similar abundances to those yellow biofilms from karstic caves were found in the 3C‐yellow, 7G‐yellow and 5E‐red biofilms from Cueva del Viento (Martin‐Pozas et al., 2023).

The high relative abundance of unidentified and uncultured taxa is noticeable in the 3C‐yellow, 7G‐yellow and 5E‐red biofilms. Similar results were reported for caves in the Galapagos Islands with an abundance of uncultured bacteria and it was suggested that lava tubes have a great potential for the isolation of novel species (Miller et al., 2020).

To reveal differences in the composition of the bacterial communities at the genus level among the different samples, a barplot is presented in Figure 6A.

We were able to discern bacteria belonging to 88 genera in common among the four microbial mats (Figure 6B). The samples with the highest number of individual unique genera in common are the 5E‐red and 7G‐yellow biofilms (32 genera), followed by 11K‐white and 7G‐yellow, sharing 25 genera. The most dissimilar samples, sharing the lowest number of individual unique genera, are the 7G‐yellow and 3C‐yellow biofilms, with three common genera. The 5E‐red and 11K‐white biofilms were found to have the highest number of unique genera (38 genera) and 3C‐yellow the lowest number (11 genera).

**FIGURE 7:**
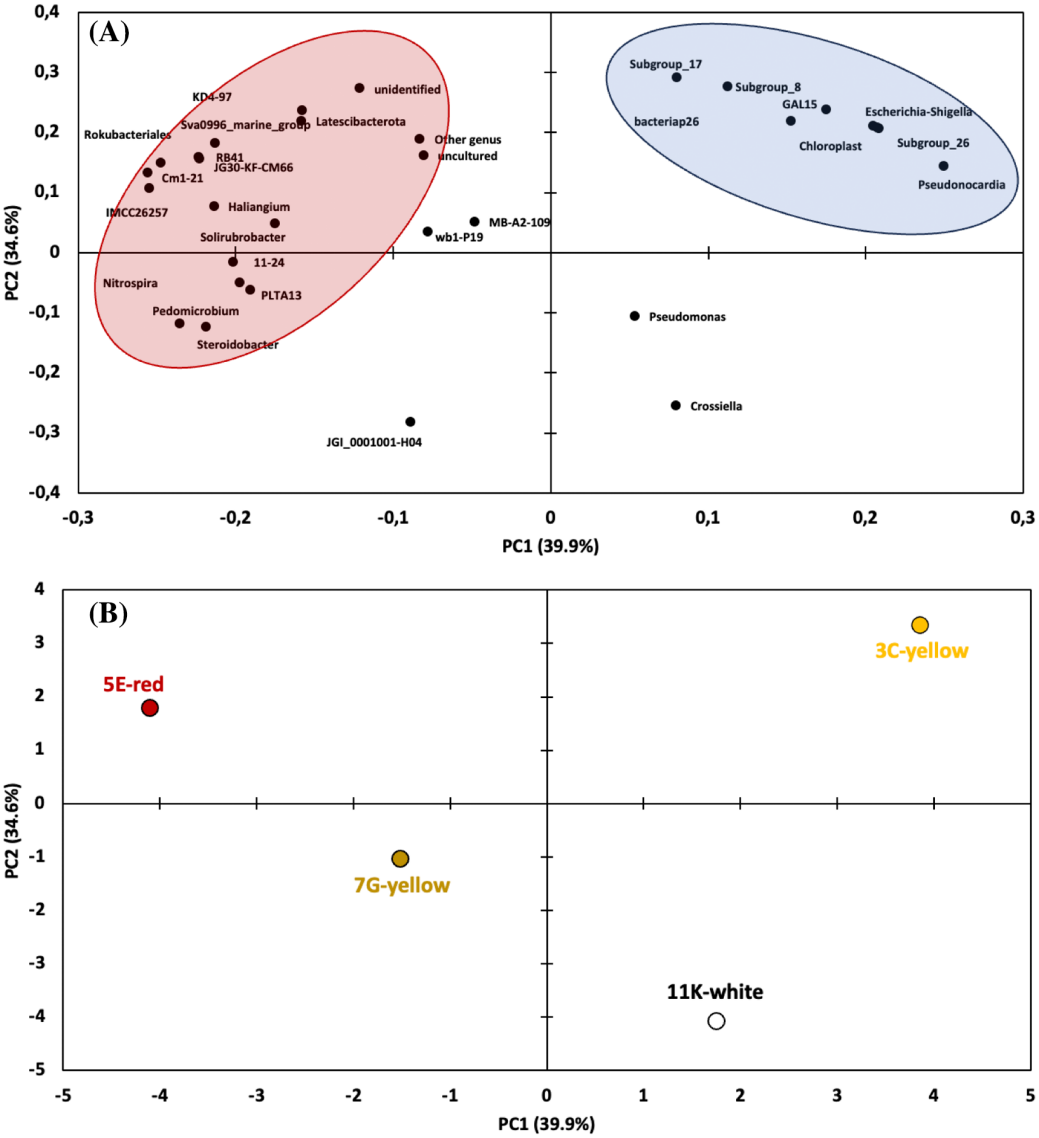
Principal component analysis (PCA). (A) loadings of variables (microbial genera); (B) Scores of the four biofilm samples.

The 5E‐red biofilm has a high similarity to the yellow biofilm based on its genus composition. However, the 11K‐white biofilm differs the most in similarity to the rest of the microbial mats based on its higher proportion (>60%) of members belonging to the *Crossiella* genus. To confirm this pattern a principal component analysis (PCA) was performed to understand the statistical relationship between the microbial diversity present in these samples (Figure 7). The analysis of the two first components (component 1: 39.9% and component 2: 34.6%) can explain the 74.5% of the total variance. The scatterplot of the loadings of PC‐1 versus PC‐2 (Figure 7A) showed the existence of two main clusters of genera. Cluster‐I is composed of a heterogeneous group of bacteria belonging to the phyla *Acidobacteriota* (11–24, RB41), *Actinomycetota* (*Solirubrobacter*, IMCC26257, and Sva0996_marine_group), *Pseudomonadota* (*Pedomicrobium*, *Steroidobacter*, Cm1.21, PLTA13), *Cloroflexota* (JG30‐KF‐CM66 and KD4‐97), *Nitrospirota* (*Nitrospira*), *Myxococcota* (*Haliangium*), *Candidatus Latescibacteria* (*Latescibacteria*) and *Candidatus* *Methylomirabilota* (*Rokubacteriales*). While cluster‐2 was composed of phylum *Acidobacteriota* (Subgroup 8, 17 and 26), *Pseudomonadota* (*Escherichia‐Shigella*), *Actinomycetota* (*Pseudonocardia*), chloroplast, GAL15 and *Myxococcota* (bacteriap26). The scores PCA plot of the biofilm samples (Figure 7B), generated using the main genera shows the existence of significant differences among samples. The 5E‐red sample is mainly dominated by the genera located in cluster I, while the genera sorted in cluster II are dominant in the 3C‐yellow sample. The 11K‐white sample is directly correlated to the genus *Crossiella*, as also observed in Figure 6A.

Second in abundance was the Subgroup 25 (*Acidobacteriota*) with 15.17% in the 3C‐yellow biofilm and lacking in the 11K‐white biofilm. The genus RB41 of *Acidobacteriota* is involved in the nitrogen cycle. This genus was found in other lava tubes (Gonzalez‐Pimentel et al., 2021; Hathaway et al., 2021). Meier et al. (2021) reported that RB41 was a key genus in N assimilation and plays a role in maintaining soil metabolism and biogeochemical function under long‐term low nutrient stress conditions. Another genus involved in the nitrogen cycle is *Nitrospira* which comprises ammonium‐oxidizing bacteria and its occurrence is ubiquitous both in volcanic (including yellow biofilms) and karstic caves (Gonzalez‐Pimentel et al., 2021; Jurado et al., 2020; Martin‐Pozas et al., 2023; Nicolosi et al., 2023; Saiz‐Jimenez et al., 2011). The genus wb1 P19 (*Pseudomonadota*) is particularly abundant in 7G‐yellow (6.87%–1.68%) and missing in the 11K‐white biofilm. This lineage is abundantly represented in most studied caves (Frazier, 2020; Jurado et al., 2020; Reboleira et al., 2022), and particularly in yellow biofilms (Bastian et al., 2009; Martin‐Pozas et al., 2023) but data on its metabolism, other than participation in the nitrogen cycle is unknown.

The occurrence of *Pseudomonas* species in caves has been related to anthropic contamination. Paradigmatic is the example of Lascaux Cave (Alonso et al., 2022; Ikner et al., 2007). Other authors also related the presence of *Pseudomonas* to the human impact on caves (Davis et al., 2020; Johnston et al., 2012; Svec et al., 2020), and a few novel species of *Pseudomonas* were isolated from caves (Busquets et al., 2021; Stomeo et al., 2008). In lava tubes from Mount Etna, frequently visited by tourists, *Pseudomonas* reached a significant abundance (Nicolosi et al., 2023).

*Pseudonocardia*, more abundant in 3C‐yellow, was also found in lava tubes from the Azores and Galapagos (Miller et al., 2020; Miller et al., 2022) and karstic caves (Lee et al., 2001). *Pseudonocardia kongjuensis* was isolated from a gold mine cave (Lavoie & Northup, 2005). *Escherichia‐Shigella*, identified in the 3C‐yellow biofilm, has been reported as an indicator of faecal contamination in caves (Bercea et al., 2019; Campbell et al., 2011; Martin‐Sanchez et al., 2015). *Escherichia*, *Shigella*, *Legionella, Inquilinus* and other pathogenic bacteria were the most representative phylotypes found in Lascaux Cave, a highly altered show cave due to wrong management in the years (Alonso et al., 2022; Fudou et al., 2002). Davis (Svec et al., 2020) found *Escherichia* after heavy rains and water runoff from the surface entering in a cave. This hypothesis could justify the presence of *Escherichia‐Shigella* and *Pseudomonas* in the 3C‐yellow biofilm from the *Sobrado Superior* section of Cueva del Viento.

*Haliangium*, found mainly in the 5E‐red biofilm, was described after isolation from marine environments of two different species, *H. ochraceum* and *H. tepidum*. The cultured members of this genus are moderately halophilic (Hu et al., 2022). The two species differ from known terrestrial myxobacteria in salt requirements and the presence of anteiso‐branched fatty acids.   *Haliangium* is a crucial species in the soil biogeochemical cycle (Hu et al., 2022). *Haliangium* has been found in a few caves (Engel et al., 2013; Park et al., 2020; Read et al., 2021; Yasir et al., 2018).

*Steroidobacter* is a relatively common genus in lava tubes (Gonzalez‐Pimentel et al., 2018; Gonzalez‐Pimentel et al., 2021; Lavoie et al., 2017; Nicolosi et al., 2023; Riquelme, Marshall Hathaway, et al., 2015; Riquelme, Rigal, et al., 2015) and identified principally in the 5E‐red biofilm. In karstic caves were also retrieved (Jurado et al., 2020; Omoregie et al., 2019; Porca et al., 2012).

**FIGURE 8:**
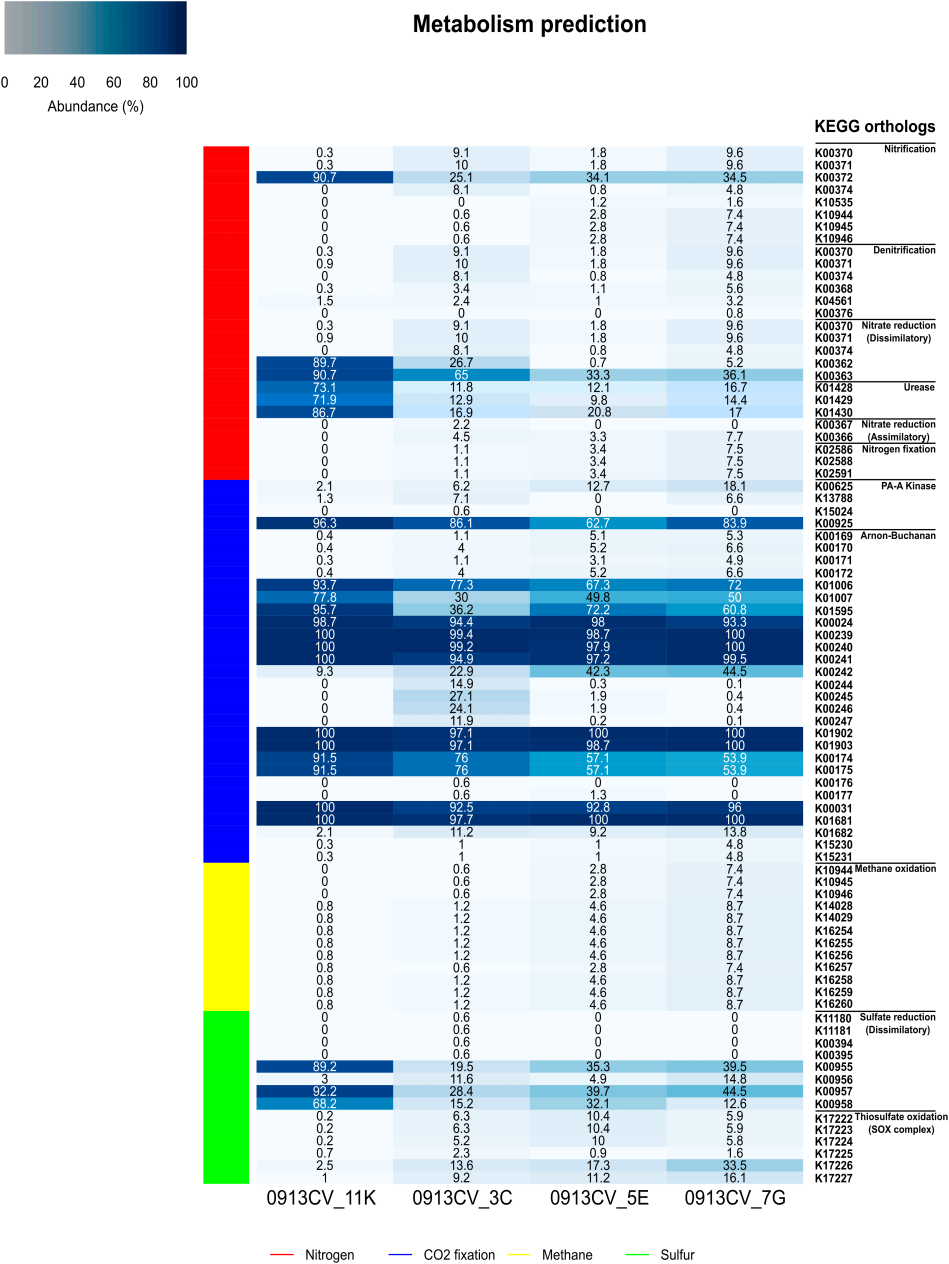
The abundance of K0 functional categories retained for KEGG pathways. Predicted metabolic pathways in prokaryotes for Nitrogen, Carbon and Sulfur cycles. Nitrogen pathways involve nitrification, denitrification, nitrogen fixation, assimilatory nitrate reduction, dissimilatory nitrate reduction and urease activity. Carbon pathways involved CO_2_ fixation (PA‐A Kinase: Phosphate acetyltransferase‐acetate kinase pathway; Arnon‐Buchanan: Reductive citrate cycle) and methane metabolism (Methane oxidation). Sulfur pathways involved dissimilatory sulfate reduction and Thiosulfate oxidation by SOX complex.

The functional profile of the bacterial communities from Cueva del Viento, as predicted by PICRUSt2, revealed a heterogeneous metabolism among samples (Figure 8). Sample 7G‐yellow was the most metabolically dynamic, while sample 11K‐white had the least influence on the biogeochemical cycle of major elements. Nitrogen pathways were widely presented in bacterial communities from samples 3C‐yellow, 5E‐red and 7G‐yellow. In sample 7G‐yellow, complete nitrification and denitrification pathways were predicted, with all K0 categories observed. The genus *Nitrospira*, present in these samples, is composed of nitrite‐oxidizing bacteria, which can be involved in the CO_2_‐fixation‐coupled ammonia oxidation process, as reported in other cave ecosystems (Sarbu et al., 1996). However, samples 3C‐yellow and 5E‐red lacked complete denitrification pathways, as they were missing the K00376 (gene nosZ), crucial for converting nitrous oxide (N_2_O) to nitrogen (N_2_). Additionally, the absence of K10535 (hydroxylamine dehydrogenase) in sample 3C‐yellow suggests an incomplete nitrification pathway.

None of the nitrogen pathways were complete for sample 11K‐white, although there was a high abundance of K00362 and K00363, genes nirB and nirD, respectively. These are directly involved in the reduction of nitrite to ammonia within the dissimilatory nitrate reduction pathway. On the other hand, K01428, K01429 and K01430 (urease enzyme structural genes) were especially abundant in the 11K‐white biofilm. In this sample, 65.63% of the identified sequences were *Crossiella*. This genus is widely distributed in soils and plant rhizospheres all over the world (Martin‐Pozas et al., 2023). It is known to produce the enzyme urease, which then catalyses the hydrolysis of urea into ammonia and carbon dioxide. The released ammonia raises the local pH, creating an alkaline environment and promoting mineral precipitation, such as calcium carbonate (Martin‐Pozas et al., 2020). The active role of *Crossiella* in biomineralization and moonmilk formation was shown by Martin‐Pozas (Martin‐Pozas et al., 2023). This demonstrates that ureolytic bacteria, such as *Crossiella* isolated from caves, are promising biomineralization agents with relevant biotechnological applications, for instance in soil biocalcification (Martin‐Pozas et al., 2020).

Focusing on the CO_2_ pathways, only the sample 3C‐yellow presented all the K0 categories for both reductive citrate cycle (Arnon‐Buchanan cycle) and the phosphate acetyltransferase‐acetate kinase pathway (PA‐A Kinase), as it was the only sample where the presence of the orthologs K15024 (putative phosphotransacetylase) and K00176 (2‐oxoglutarate ferredoxin oxidoreductase subunit delta), involved in the PA‐A Kinase pathway and the Arnon‐Buchanan cycle, respectively, were predicted.

The methane metabolism, through the methane oxidation pathway, was predicted in samples 3C‐yellow, 5E‐red and 7G‐yellow, with the latter showing the orthologs involved to be more abundant, ranging from 7.4% to 8.7%. Sample 5E‐red presented 2.8%–4.6%, whereas the 3C‐yellow biofilm ranged between 0.6% and 1.2%. Martín‐Pozas reported that methane‐oxidizing bacteria found in caves consume methane that enters into the underground atmosphere and produces bioactive compounds (Martin‐Pozas et al., 2020).

Concerning sulfur metabolism, the prediction of K0 orthologs indicated that 3C‐yellow had a more active metabolism. This was evidenced by the presence of necessary orthologs involved in both dissimilatory sulfate reduction and thiosulfate oxidation via the SOX complex pathways (Figure 8). This functional capacity of the bacterial communities dwelling in the Cueva del Viento lava tube system is in line with recent studies carried out in lava tubes from the Canary Islands, specifically from La Palma (Gonzalez‐Pimentel et al., 2021) and Lanzarote (Palma et al., 2024), suggesting a determinant role of the microorganisms from the Canary lava tubes in the biogeochemical cycling of major elements.

Traditional approaches used to study the diversity of microbial communities in oligotrophic environments are challenging. Although advances in NGS technologies in the last decade have enabled the study of complete microbiomes, both approaches should be used in parallel to provide a deeper understanding and exploitation of the microbial diversity associated with these poorly studied ecosystems. In this study, we reported differences in the identification of microbial groups when we compared the diversity obtained using both techniques. As culture‐dependent techniques involve growing microorganisms on specific nutrient media to provide the necessary conditions for their growth, it is not easy to replicate the environmental conditions, such as temperature, pH and nutrient availability, specific for their growth, as in Cueva del Viento. Moreover, culture techniques may favour the growth of certain fast‐growing or dominant microbial species, leading to an underrepresentation of the overall microbial diversity. The large number of isolates belonging to the phyla *Actinomycetota* and *Bacillota* found in these biofilms can be explained by the fact that most of their members produce spores and can grow optimally in the culture media used in the laboratory (Lee et al., 2001). However, it enabled obtaining pure cultures to study their morphological and potential metabolic characteristics. The NGS technologies allowed us to obtain a comprehensive vision of the bacterial communities present and the estimation of the bacterial richness, diversity and functional profiles of the studied lava tube samples. Considering the inhospitable and unique characteristics of the ecosystem under study, the isolated species will be subjected to further investigation to assess their antimicrobial properties and susceptibility to antibiotics or other substances, as conducted by Riquelme (Riquelme et al., 2017). These tests can hold significant potential for various biotechnological applications, including but not limited to the pharmaceutical and agricultural industries.

## CONCLUSIONS

The microbial communities of the different microbial mats from Cueva del Viento are similar in terms of phyla distribution to other lava tubes previously reported, and particularly noticeable are the similarities among yellow biofilms from different geographical regions. Three phyla, *Actinomycetota, Pseudomonadota* and *Acidobacteriota* dominated the four sampling sites. One of the most remarkable facts is the high relative abundance of the genus *Crossiella* in the biofilms, which agrees with previous data from different volcanic and karstic caves, pointing out the presence of biomineralization processes associated with the white microbial mats from Cueva del Viento. Also notable is the occurrence of genera involved in the nitrogen cycle (RB41 and *Nitrospira*). The high abundance of uncultured and unassigned taxa in the biofilms is relevant and points to the occurrence of abundant microbial dark matter in this cave.

In this sense, lava tubes, such as the Cueva del Viento volcanic system, can be a good setting for studying microbial diversity, characterizing new bacterial species, and studying the production of bioactive substances, especially in the *Streptomyces*, *Paenarthrobacter* and *Pseudomonas* genera. Indeed, the strain identified as *Pseudomonas yangonensis* presented a special interest because it possessed low similarity percentages (97.89%) concerning the 16S rRNA gene sequences available in the EzTaxon databases. The homology of the complete 16S rRNA sequence is one of the first data to be considered for the selection and study of possible new species. For this reason, other strains isolated from lava tubes should be investigated in future taxonomic studies.

Our study emphasizes that lava tubes provide unique and extreme habitats for microbial life, which can thrive and interact with siliceous minerals, as observed in the Cueva del Viento volcanic system. The metabarcoding approach provided a broader perspective of the microbial diversity of the Cueva del Viento lava tube system by directly analysing DNA from the environmental samples. A combination of both approaches allowed obtaining a more comprehensive understanding of cave microbial communities. These results highlight the need for further study on the microbial diversity of lava tubes and associated geomicrobiological interactions through targeted culturing methods and metagenomics, combined with electron microscopy techniques. Such interdisciplinary efforts will allow us to have a better understanding of the processes that occur in subterranean environments and the measures that should be taken for their conservation and protection.

## AUTHOR CONTRIBUTIONS

**Sara Gutierrez‐Patricio:** Data curation (equal); formal analysis (lead); methodology (equal); writing – original draft (lead); writing – review and editing (equal). **Jorge Osman:** Formal analysis (equal); writing – review and editing (equal). **José Luis Gonzalez‐Pimentel:** Formal analysis (equal); software (lead); writing – review and editing (equal). **Valme Jurado:** Investigation (equal); methodology (equal); supervision (equal); validation (equal); writing – review and editing (equal). **Leonila Laiz:** Investigation (equal); methodology (equal); supervision (equal); validation (equal); writing – review and editing (equal). **Alfredo Laínez Concepción:** Investigation (equal); visualization (equal); writing – review and editing (equal). **Cesareo Saiz‐Jimenez:** Data curation (equal); investigation (equal); methodology (equal); supervision (lead); writing – original draft (lead); writing – review and editing (equal). **Ana Zélia Miller:** Conceptualization (equal); data curation (equal); funding acquisition (lead); investigation (equal); methodology (equal); supervision (lead); writing – review and editing (lead).

## CONFLICT OF INTEREST STATEMENT

The authors declare no conflict of interest.

## Supporting information


**TABLE S1.** Bacterial strains isolated from the microbial mats collected in *Cueva del Viento* lava tube system.

## Data Availability

The raw reads were deposited into the NCBI Sequence Read Archive (SRA) database under BioProject PRJNA914266.
