# Supplementary material for: Microbiological exploration of the Cueva del Viento lava tube system in Tenerife, Canary Islands
Source: Environ Microbiol Rep. 2024 Apr 21;16(2):e13245. doi: 10.1111/1758-2229.13245 (PMC11033209; doi:10.1111/1758-2229.13245)
Supplement: Supplementary file 1 — TABLE S1. Bacterial strains isolated from the microbial mats collected in Cueva del Viento lava tube system. [file EMI4-16-e13245-s001.docx]

**Suplementary table 1**: Bacterial strains isolated from the microbial mats collected in *Cueva del Viento* lava tube system.

| **Sample** | **Strain name** | **Access Number** | **Phylum** | **Genera** | **Identification** | **Similarity (%)** |
| --- | --- | --- | --- | --- | --- | --- |
| 3C-Yellow | 0913CV3CTS3 | LN867256 | *Actinomicetota* | *Streptomyces* | *Streptomyces benahoarensis* (VKJP01000401) | 100 |
| 3C-Yellow | 0913CV3CTS5 | LN867257 | *Actinomicetota* | *Streptomyces* | *Streptomyces rhizosphaerihabitans* (HQ267983) | 98.75 |
| 3C-Yellow | 0913CV3CT5 | LN867258 | *Actinomicetota* | *Streptomyces* | *Streptomyces* sp.^1^ | 100 |
| 3C-Yellow | 0913CV3CTS4 | LN867259 | *Actinomicetota* | *Streptomyces* | *Streptomyces apricus* (MN133488) | 98.91 |
| 3C-Yellow | 0913CV3CT2 | LN867259 | *Actinomicetota* | *Streptomyces* | *Streptomyces benahoarensis* (VKJP01000401) | 99.89 |
| 3C-Yellow | 0913CV3CTS6 | LN867261 | *Actinomicetota* | *Streptomyces* | *Streptomyces* sp.^2^ | 99.17 |
| 3C-Yellow | 0913CV3CT1 | LN867262 | *Bacillota* | *Bacillus* | *Bacillus* sp.^3^ | 100 |
| 3C-Yellow | 0913CV3CNS1,2 | LN867263 | *Bacillota* | *Peribacillus* | *Peribacillus frigoritolerans* (AM747813)/*Peribacillus simplex* (BCVO01000086) | 99.32 |
| 5E-Red | 0913CV5ET4 | LN867264 | *Actinomicetota* | *Streptomyces* | *Streptomyces* sp.^4^ | 99.78 |
| 5E-Red | 0913CV5ET7 | LN867265 | *Bacillota* | *Bacillus* | *Bacillus atrophaeus* (AB021181) | 99.77 |
| 5E-Red | 0913CV5ETS4 | LN867266 | *Bacillota* | *Bacillus* | *Bacillus siamensis* (AJVF01000043) | 99.29 |
| 5E-Red | 0913CV5ET2 | LN867267 | *Bacillota* | *Bacillus* | *Bacillus* sp.^5^ | 99.85 |
| 5E-Red | 0913CV5ENS2,2 | LN867268 | *Bacillota* | *Bacillus* | *Bacillus tequilensis* (AYTO01000043) | 100 |
| 5E-Red | 0913CV5ETS6 | LN881695 | *Bacillota* | *Bacillus* | *Bacillus tequilensis* (AYTO01000043) | 99.19 |
| 5E-Red | 0913CV5ENS2,1 | LN881696 | *Bacillota* | *Bacillus* | *Bacillus tequilensis* (AYTO01000043) | 99.08 |
| 7G-Yellow | 0913CV7GTS6 | LN867269 | *Actinomicetota* | *Paenarthrobacter* | *Paenarthrobacter nicotinovorans* (X80743) | 99.41 |
| 7G-Yellow | 0913CV7GNS1 | LN881697 | *Actinomicetota* | *Paenarthrobacter* | *Paenarthrobacter nicotinovorans* (X80743) | 99.35 |
| 7G-Yellow | 0913CV7GTS4 | LN881698 | *Actinomicetota* | *Paenarthrobacter* | *Paenarthrobacter nicotinovorans* (X80743) | 99.12 |
| 7G-Yellow | 0913CV7GN7,1 | LN867270 | *Actinomicetota* | *Micrococcus* | *Micrococcus luteus* (CP001628) | 99.12 |

**Suplementary table 1 (Cont.)**

| **Sample** | **Strain name** | **Access Number** | **Phylum** | **Genera** | **Identification** | **Similarity (%)** |
| --- | --- | --- | --- | --- | --- | --- |
| 7G-Yellow | 0913CV7GTS1,2 | LN867271 | *Actinomicetota* | *Micrococcus* | *Micrococcus luteus* (CP001628) | 99.87 |
| 7G-Yellow | 0913CV7GNS2 | LN867272 | *Bacillota* | *Peribacillus* | *Peribacillus simplex* (BCVO01000086) | 99.80 |
| 7G-Yellow | 0913CV7GTS8 | LN881699 | *Bacillota* | *Peribacillus* | *Peribacillus simplex* (BCVO01000086) | 99.66 |
| 7G-Yellow | 0913CV7GTS2 | LN867273 | *Bacillota* | *Bacillus* | *Bacillus siamensis* (AJVF01000043) | 99.80 |
| 7G-Yellow | 0913CV7GN6 | LN867274 | *Bacillota* | *Bacillus* | *Bacillus mycoides* (ACMU01000002) | 99.77 |
| 7G-Yellow | 0913CV7GT7,2 | LN867275 | *Bacillota* | *Rossellomorea* | *Rossellomorea vietnamensis* (AB099708) | 99.58 |
| 7G-Yellow | 0913CV7GT7,1 | LN881700 | *Bacillota* | *Rossellomorea* | *Rossellomorea vietnamensis* (AB099708) | 99.36 |
| 7G-Yellow | 0913CV7GT5 | LN867276 | *Pseudomonadota* | *Pseudomonas* | *Pseudomonas laurylsulfatiphila* (KY462012) | 99.40 |
| 7G-Yellow | 0913CV7GTS5 | LN867277 | *Bacillota* | *Psychrobacillus* | *Psychrobacillus soli* (KJ956929) | 99.42 |
| 11K-White | 0913CV11KT10 | LN867278 | *Actinomicetota* | *Arthrobacter* | *Arthrobacter ginkgonis* (KP128918) | 99.21 |
| 11K-White | 0913CV11KTS3 | LN867279 | *Actinomicetota* | *Paenarthrobacter* | *Paenarthrobacter nicotinovorans* (X80743) | 99.42 |
| 11K-White | 0913CV11KN7 | LN867280 | *Actinomicetota* | *Paenarthrobacter* | *Paenarthrobacter nicotinovorans* (X80743) | 99.38 |
| 11K-White | 0913CV11KT5 |  | *Actinomicetota* | *Micrococcus* | *Micrococcus luteus* (CP001628) | 99.52 |
| 11K-White | 0913CV11KTS6 | LN867281 | *Actinomicetota* | *Micrococcus* | *Micrococcus luteus* (CP001628) | 99.58 |
| 11K-White | 0913CV11KTS1 | LN867282 | *Actinomicetota* | *Streptomyces* | *Streptomyces* sp.^4^ | 99.46 |
| 11K-White | 0913CV11KTS2 | LN867283 | *Bacillota* | *Sutcliffiella* | *Sutcliffiella halmapala* (KV917375) | 99.23 |
| 11K-White | 0913CV11KTS5 | LN867284 | *Bacillota* | *Metabacillus* | *Metabacillus rhizolycopersici* (OK274256) | 100 |
| 11K-White | 0913CV11KT6 | LN867285 | *Pseudomonadota* | *Pseudomonas* | *Pseudomonas yangonensis* (MK907288) | 97.89 |
| 11K-White | 0913CV11KN6,1 |  | *Pseudomonadota* | *Pseudomonas* | *Pseudomonas yangonensis* (MK907288) | 98.59 |
| 11K-White | 0913CV11KNS1 | LN867286 | *Pseudomonadota* | *Pseudomonas* | *Pseudomonas lalkuanensis* (MF943158) | 98.87 |
| 11K-White | 0913CV11KN6,2 | LN867287 | *Pseudomonadota* | *Pseudoxanthomonas* | *Pseudoxanthomonas* sp.^6^ | 99.38 |
| 11K-White | 0913CV11KN3,2 | LN867288 | *Bacteroidota* | *Chryseobacterium* | *Chryseobacterium candidae* (SDLV01000037) | 99.32 |
| 11K-White | 0913CV11KN2 | LN867289 | *Bacteroidota* | *Flavihumibacter* | *Flavihumibacter fluminis* (OM018663) | 98.64 |

^1^ Share the same similarity with *Streptomyces cyaneus* (AF346475), *S. lincolnensis* (X79854), *S. antibioticus* (AB184184), *S. mirabilis* (AB184412), *S. shaanxiensis* (FJ465151) and *S. griseoruber* (AB184209).

^2^ Taxonomic group *Streptomyces lydicus*: *S. lydicus* (AB184281) and *S. chattanoogensis* (AJ621611).

^3^ Taxonomic group *Bacillus mycoides*: *B. mycoides* (ACMU01000002) and *B. weihenstephanensis* (BAUY01000093).

^4^ Taxonomic group *Streptomyces lavendulae*: *Streptomyces avidinii (*AB184395), *Streptomyces xanthophaeus* (AB184177), *Streptomyces cirratus* (AY999794), *Streptomyces vinaceus* (AB184394), *Streptomyces spororaveus* (AJ781370), *Streptomyces nojiriensis* (AJ781355), *Streptomyces lavendulae subsp. lavendulae* (AB184146), among others.

^5^ Share the same similarity with: *Bacillus zhangzhouensis* (JOTP01000061), *Bacillus safensis subsp. safensis* (ASJD01000027) and *Bacillus safensis subsp. osmophilus* (KY990920).

^6^ Share the same similarity with *Pseudoxanthomonas mexicana* (AF273082), *P. japonensis* (AB008507) and *P. arseniciresistens* (MN685193).
